# Risk factors associated with hypertensive disorders in pregnancy in Nekemte referral hospital, from July 2015 to June 2017, Ethiopia: case-control study

**DOI:** 10.1186/s12884-019-2693-9

**Published:** 2020-01-06

**Authors:** Leta Hinkosa, Almaz Tamene, Negeso Gebeyehu

**Affiliations:** 1 Department of Midwifery, Institute of Health Sciences , Wollega University, Nekemte, Ethiopia; 2 Nekemte Referral Hospital, Oromia Regional National State, Nekemte, Ethiopia; 3 Department of Midwifery, College of Medicine and Health Sciences, Wechamo University, Hossana, Ethiopia

**Keywords:** Risk factors, Hypertension disorders, Pregnancy

## Abstract

**Background:**

Hypertension is the most common medical problem encountered in pregnancy and is a leading cause of perinatal and maternal morbidity and mortality. However, its magnitude and risk factors yet not adequately assessed at the study area.

**Methods:**

Facility-based retrospective unmatched case-control study was conducted to identify risk factors associated with Hypertensive disorders of pregnancy in Nekemte Referral Hospital just two years back from study period July 1, 2015, to June 30, 2017. Bivariate logistic regression was considered for inclusion in to the multivariate logistic regression. Finally, multi varaite analysis were done to identify risk factors of hypertensive disorders of pregnancy.

**Results:**

Among 6826 total delivery records from July 2015 –June 2017, 199 women developed hypertension during pregnancy. Among 199 women 153(76.9%) were pre-eclampsia/eclampsia,28(14.1%) were gestational hypertension, 14(0.7%) were superimposed hypertension and 4 (2.9%) were chronic hypertension.

Age ≥ 35 (AOR: 2.51, 95% CI: 1.08, 5.83), rural residential area (AOR: 1.79, 95% CI: 1.150, 2.799), prim gravida (AOR: 3.39, 95% CI: 2.16, 5.33), null parity (AOR: 4.35, 95% CI: 2.36, 8.03), positive history of abortion (AOR: 4.39, 95% CI: 1.64, 11.76), twin pregnancy (AOR: 3.78, 95% CI: 1.52, 9.39), lack of ANC follow up (AOR: 3.05, 95% CI: 1.56, 5.96) as well as positive pre-existing hypertension (AOR: 3.81, 95% CI: 1.69, 8.58), positive family history of hypertension (AOR: 5.04, 95% CI: 2.66, 9.56) and positive history of diabetes mellitus (AOR: 5.03, 95% CI: 1.59, 15.89) were risk factors for hypertensive disorders during pregnancy.

**Conclusion:**

This study found that Women with hypertension during pregnancy have a greater risk of developing adverse pregnancy outcome as compared to normotensive pregnant women. so, identification of these risk factors would be useful for early diagnosis of hypertension disorders during pregnancy to give appropriate clinical monitoring and treatments and timely managing maternal and perinatal complications.

## Background

Hypertension is a clinical term used to describe high blood pressure [1, 2]. Hypertension in pregnancy is defined as: “Systolic blood pressure greater than or equal to 140 mmHg and/or diastolic blood pressure greater than or equal to 90 mmHg which usually confirmed within four hours apart measurement” [2].

Hypertension disorder of pregnancy encompasses a spectrum of conditions including pre-existing hypertension, gestational hypertension, preeclampsia/eclampsia, and superimposed hypertension.

These conditions range from a mild increase in blood pressure at term with no additional signs or symptoms to severe complications with potential for significant maternal, fetal and neonatal harm [3]. Globally, a significant number of women die every year from pregnancy-related causes and more than half of these deaths occur in sub-Saharan Africa [4]. Approximately 12% of the maternal deaths are associated with hypertensive disorders in pregnancy such as pregnancy-induced hypertension [1–4,]. For that reason, hypertension complications are among the main public health issues worldwide.

A Hospital-based cross-sectional study conducted in Jimma University Specialized Hospital in Ethiopia showed that the overall prevalence of hypertensive disorders of pregnancy was 8.5% of which severe preeclampsia and eclampsia accounted for 51.9 and 23.4%, respectively [5]. Moreover, the study done in Debre Brehan Referral Hospital indicated that among 8626 pregnant women who obtained delivery services, 340(3.9%) of them had hypertensive disorders with an increasing trend from 1.8% in 2011 to 5.7% in 2014 [6].

On the other hand, though in Ethiopia, the efforts have been done to identify the risk factors of hypertension and to overcome its effect, its prevalence and risk factors were increasing in the country. The study area population was found in the western part of the country. The study area was the place where the population was highly affected by hypertension disorders during pregnancy. Besides, there is a scarcity of study conducted on risk factors associated with HDP in Nekemte Referral Hospital.

Therefore, it is essential to undertake this study to determine the risk factors and its complications both on mothers and on new-borne in the Hospital.

## Methods

The facility-based retrospective case-control study was conducted to identify risk factors associated with hypertensive disorders of pregnancy in Nekemte Referral Hospital from July 1, 2015, to June 30, 2017.

### Source population

All mothers who delivered in Nekemte Referral hospital.

### Study population

#### Case group

Cards of mothers who gave birth in Nekemte referral hospital from of July 2015 to June 2017 and found to have hypertensive disorders during pregnancy.

#### Control group

Cards of mothers who gave birth in the hospital and not identified to have hypertensive disorders during pregnancy.

### Sample size determination

The sample size was determined on the assumptions of the ratios of 1:2, (cases to controls) power 80%, alpha value 95%, and odd ratio 2 by considering relevant factors from other studies that have significant association with hypertension [7–9, and].

**From** Table 1, The final sample size was taken from diabetes by adding 10% for incomplete record reviews for the control group since it is the maximum for case (243) and for control (534) the total sample size was 777.

**Table 1:** Sample size determination

Relevant factors	Expected frequency of exposure among control	OR	Case	Control	Total Sample
Diabetes	9%	2.4	243	485	728
Prime gravid	28.08%	2.1–3	116	232	348
Age (> 35 years old)	31.3%	4.5	136	272	408

### Sampling technique and procedure

Among 6826 records of pregnant mothers who gave birth in the study areas, from July 2015 to June 2017 were first sorted for hypertension and without hypertension. Then, based on 1:2 ratios of samples of cases and controls, respectively, 534 (including 10% for incomplete records) normotensive deliveries were randomly selected.

Out of 777 selected records, 44 cases and 136 controls were excluded from analysis for incomplete of the necessary information. The final data of the study were collected from 199 (81.9%) cases and 398 (74.5%) controls which adds to up 597 women by inclusion criteria.

### Variables of the study

A. **Dependent variable**: Hypertensive disorder of pregnancy.

B. **Independent variables.**

**Demographic variables**: Age, Residential area, Marital status, Plan of pregnancy.

**Obstetric factors**: Gravida. Parity. Abortion history, ANC follow up, Multiplicity of pregnancy.

**Medical Disease factors**: Pre-existing hypertension, Family history of hypertension. History of diabetes mellitus.

### Data collection

Data was collected from record review using a structured and pre-tested checklist. The training was given for both data collectors and supervisors. Three midwives were assigned to collect the data, one supervisor was assigned to supervise the quality data collection.

### Data analysis procedures

Records that shows hypertension during pregnancy were taken as case group and the remaining registries were taken as a control group. Then, to identify the sample and control group, the medical record was retrieved and checked for hypertension during pregnancy.

Accordingly, records that show one of the four of HDP types (gestational hypertension, chronic hypertension, pre-eclampsia/ eclampsia or superimposed hypertension) were taken as case group first and then from the remaining registries control group was randomly obtained.

The criteria were an elevation of blood pressure for gestational hypertension whereas blood pressure, protein urea and other laboratory investigations were used as a criterion for other types of hypertensive disorders. Then finally descriptive statics and logistic regression were used. Descriptive statistics such as frequency, a measure of central tendency and measure of dispersion where were calculated to describe the study sample and presented with tables and figures. To determine factors that were significantly associated with hypertension, the first bivariate logistic regression was done. Then, multiple logistic analysis was performed for those variables identified as significant on bivariate analysis.

## Results

Among 6826 of the total delivery records during the study period, 243 (3.56%) women had HDP. Of 777 selected records, 44 cases and 136 controls were excluded from analysis for incomplete of necessary information. The final data of the study were collected from 199 (81.9%) of cases and 398 (74.5%) of controls which adds to up 597 women.

### Demographic characteristics of women with and without HDP

The mean age of cases was 26.1(SD: ±6.1) which was higher than that of the controls 24.4(SD: ±4.9). Eighty-six (43.2%) of the cases and 218(54.8%) of the controls were below the age of 25 years whereas 28(14.1%) of the cases and 19(4.8%) of the controls were above the age of 35 years **(**Table 2**).**

**Table 2:** Demographic characteristics of women with and without Hypertension disorders of pregnancy attended delivery service in the year July 2015–June 2017

Variables	Case	Control	*X*^*2*^	*P*-value
	Number (%)	Number (%)		
Age category				
25–29	54 (27.1)	113 (28.4)	19.36	.000
< 25	86 (43.2)	218 (54.8)		
30–34	31 (15.6)	48 (12.1)		
> 34	28 (14.1)	19 (4.8)		
Mean (SD)	26.1 (6.1)	24.4 (4.9)		
Residence				
Urban	70 (35.2)	183 (46.0)	5.91	.015
Rural	129 (64.8)	215 (54.0)		
Marital status				
Single	22 (11.1)	20 (5.0)	6.48	.011
Married	177 (88.9)	378 (95.0)		
Pregnancy plan				
Wanted	179 (89.9)	374 (94.0)	2.58	.108
Unwanted	20 (10.1)	24 (6.0)		

### Medical disease history of women with and without HDP

Concerning to medical disease factors, 35(17.6%) of cases and 15(3.8%) of controls had positive pre-existing hypertension whereas 164(82.4%) of cases and 383(96.2%) of controls had not pre-existing hypertension.

### Obstetric history characteristics of women with and without HDP

Among study participants, 109 (54.8%) of the case group and 102(25.6%) of the control group were identified for prim gravida while 90(45.2%) cases and 296(74.4%) of controls were of multigravida pregnancies **(**Table 3**).** Regarding parity, 64(32.2%) of cases and 24(6.0%) of controls were found to be nulliparous whereas 135(67.8%) of cases and 374(94.0%) controls were of parity greater or equal to 1. The parity difference between two groups was significant (χ2 (1, *n* = 597) =70.02, *p* = .00, phi = −.35).

**Table 3:** Obstetric history of women with and without Hypertension disorders attended for delivery service in the year July 2015–June 2017

Variables	Case	Control	*X*^*2*^	P-value
	Number (%)	Number (%)		
Current pregnancy history: Gravida				
Prim gravida	109 (54.8)	102 (25.6)	48.05	.000
Multigravida	90 (45.2)	296 (74.4)		
Current pregnancy history: Parity				
Null parity	64 (32.2)	24 (6.0)	70.02	.000
Parity > or = 1	135 (67.8)	374 (94.0)		
Current pregnancy history: Abortion				
No	166 (83.4)	390 (98.0)	41.80	.000
Yes	33 (16.6)	8 (2.0)		
Multiplicity of pregnancy				
Single	181 (91.0)	384 (96.5)	6.94	.008
Twin/Multiple	18 (9.0)	14 (3.5)		
ANC follow up history				
No	40 (20.1)	23 (5.8)	27.33	.000
Yes	159 (79.9)	375 (94.2)		

**Fig. 1:**
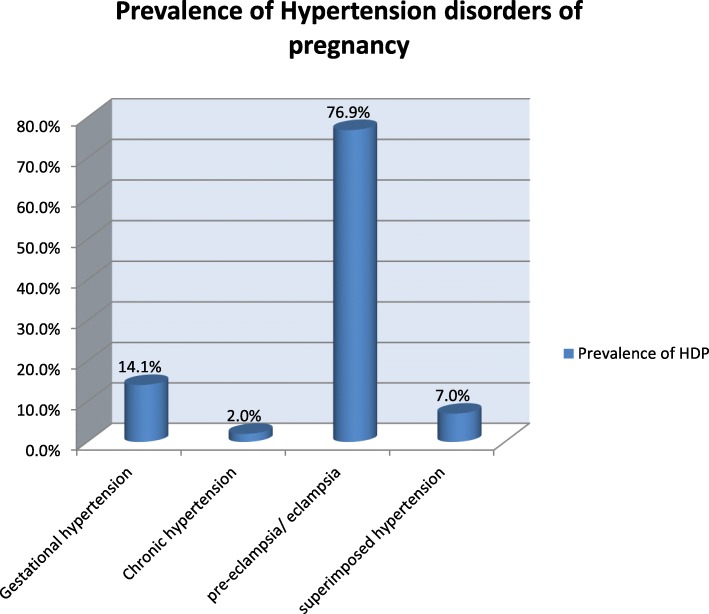
Percentage of Prevalence of Hypertension disorders among cases of the study

As it showed in Fig. 1: there were 153 (76.9%) pre-eclampsia/ eclampsia, 28 (14.1%) gestational hypertension, 14(7.0%) superimposed hypertension, and 4(2.0%) chronic hypertension (Fig. 1).

### Multivariate analysis of risk factors for women with and without HDP

The multivariate analysis revealed that, the age category of 35 years and above (AOR: 2.51, 95% CI: 1.08, 5.83), rural dwellers (AOR: 1.79, 95% CI: 1.15, 2.80), prim gravida pregnancies (AOR: 3.39, 95% CI: 2.16, 5.33),null parity (AOR: 4.35, 95% CI: 2.36, 8.03),women who had positive history of abortion (AOR: 4.39, 95% CI: 1.64, 11.76),Twin pregnancies (AOR: 3.78, 95% CI: 1.52, 9.39), ANC follow up (AOR: 3.05, 95% CI: 1.56, 5.96), positive pre-existing history of hypertension (AOR: 3.81, 95% CI: 1.69, 8.58),family history of hypertension (AOR: 5.04, 95% CI: 2.66, 9.56) History of diabetes mellitus (AOR: 5.03, 95% CI: 1.59, 15.89) were risk factors for hypertension disorders during pregnancy **(**Table 4**).**

**Table 4:** Multivariate analysis of risk factors for mothers attended delivery services in July 2015–June 2017

Variables	Case	Control	COR (95%: CI)	AOR (95%:CI)
	Number (%)	Number (%)		
Age category				
25–29	54 (27.1) 86 (43.2)	113 (28.4)	1	1
< 25	31 (15.6)	218 (54.8)	.826(.548, 1.243)	.744(.446, 1.240)
30–34	28 (14.1)	48 (12.1)	1.351(.775, 2.356)	1.020(.513, 2.026)
> 34		19 (4.8)	3.084 (1.583, 6.007) **	2.508 (1.078, 5.832) *
Residential				
Urban	70 (35.2) 129 (64.8)	183 (46.0) 215 (54.0)	1	1
Rural			1.569 (1.104,2.229) *	1.794 (1.150, 2.799) *
Marital status				
Single	22 (11.1)	20 (5.0)	2.349 (1.250, 4.417) **	1.400(.588, 3.330)
Married	177 (88.9)	378 (95.0)	1	1
Gravida				
Prim gravida	109 (54.8)	102 (25.6)	3.515 (2.456, 5.030) **	3.392 (2.159, 5.330) **
Multigravida	90 (45.2)	296 (74.4)	1	1
Parity				
Null parity	64 (32.2)	24 (6.0)	7.388 (4.442, 12.287) **	4.349 (2.355, 8.032) **
Parity > or = 1	135 (67.8)	374 (94.0)	1	1
Abortion				
No	166 (83.4)	390 (98.0)	1	1
Yes	33 (16.6)	8 (2.0)	9.691 (4.383, 21.428) **	4.390 (1.639, 11.761) **
Multiplicity of pregnancy				
Single	181 (91.0)	384 (96.5)	1	1
Twin/Multiple	18 (9.0)	14 (3.5)	2.728 (1.327, 5.606) **	3.777 (1.520, 9.387) **
ANC follow up				
No	40 (20.1)	23 (5.8)	4.102 (2.377, 7.077) **	3.048 (1.560, 5.958) **
Yes	159 (79.9)	375 (94.2)	1	1
Pre-existing hypertension				
No	164 (82.4)	383 (96.2)	1	1
Yes	35 (17.6)	15 (3.8)	5.449 (2.897, 10.251) **	3.805 (1.687, 8.581) **
Family history of hypertension				
No	137 (68.8)	373 (93.7)	1	1
Yes	62 (31.2)	25 (6.3)	6.752 (4.079, 11.176) **	5.044 (2.663, 9.555) **
History of diabetes mellitus				
No	187 (94.0)	392 (98.5)	1	1
Yes	12 (6.0)	6 (1.5)	4.193 (1.550, 11.343) **	5.032 (1.594, 15.891) **

### Differences in maternal outcomes between women with and without HDP

Regarding the onset of labor, induced labor or C/S 108(54.3%) for cases and 48(12.1%) controls. The difference of onset of labor between those with and without HDP groups was significant (χ2 (1, *n* = 597) = 123.50, *p* = .000, phi = .46) **(**Table 5**).**

**Table 5:** Differences in Pregnancy outcomes between women with and without Hypertension disorders attended delivery services in the year July 2015–June 2017

Variables	Case (%)	Control (%)	*X*^*2*^	p
Onset of labor				
Spontaneous	88 (44.2)	345 (87.9)	123.50	.000
Induced labor or c/s	108 (54.3)	48 (12.1)		
Unspecified	3 (1.5)	5 (1.3)		
Maternal death				
No	187 (94.9)	390 (98.0)	8.05	.018
Yes	9 (4.5)	4 (1.0)		
Unspecified	3 (1.5)	4 (1.0)		
Abruptio placenta				
No	177 (88.9)	388 (97.5)	28.91	.000
Yes	19 (9.5)	3 (0.8)		
Unspecified	3 (1.5)	7 (1.8)		
Preterm delivery				
No	117 (58.8)	386 (96.9)	154.24	.000
Yes	80 (41.2)	8 (2.0)		
Unspecified	2 (1.0)	4 (1.0)		

Normal and instrumental deliveries were higher among controls (60.8%) and (44.2%) than cases (29.9%) and (16.1%) respectively.

### Differences in perinatal outcomes between women with and without HDP

**Fig. 2:**
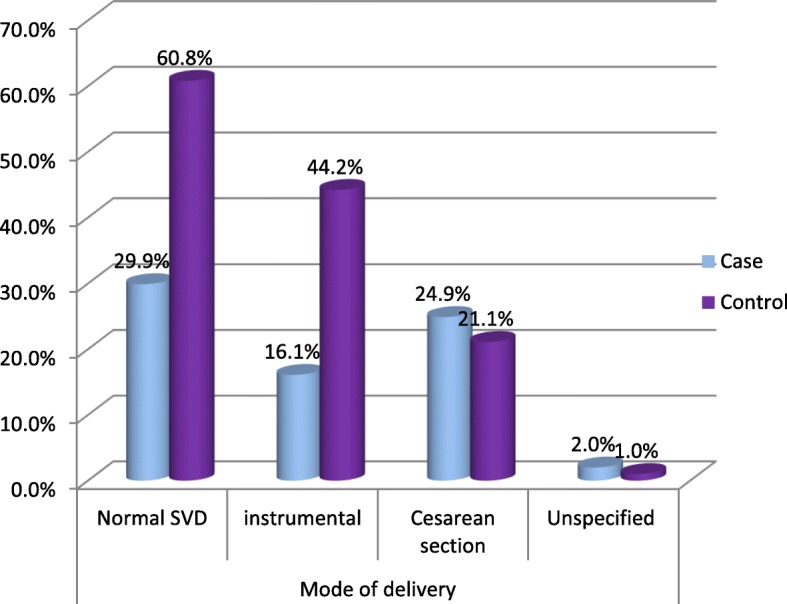
Delivery modes for women with and without Hypertension disorders of pregnancy

There was low birth weight for 72(36.2%) cases and 15(3.8%) controls (Fig. 2).

The difference of number of low birth weight between both groups was significant (χ2 (1, *n* = 597) = 123.76, *p* = .000, phi = .46) **(**Table 6**.)**

**Table 6:** Different in perinatal outcome among women with Hypertension disorders of pregnancy

Variables	Case	Control	*X*^*2*^	p
Low birth Weight				
No	121 (60.0)	382 (96.0)	123.76	.000
Yes	72 (36.2)	15 (3.8)		
Unspecified	6 (3.0)	1 (0.3)		
Stillbirth				
No	149 (74.9)	376 (94.5)	48.97	.000
Yes	44 (22.1)	21 (5.3)		
Unspecified	6 (3.0)	1 (0.3)		
Admission to NICU				
No	123 (61.8)	332 (83.4)	8.05	.018
Yes	58 (29.1)	33 (8.3)		
Unspecified	18 (9.0)	33 (8.3)		

## Discussion

### Risk factors of hypertension disorders during pregnancy

This study was conducted to identify the possible risk factors, maternal and perinatal outcomes of hypertensive disorders in pregnancy in Nekemte Referral Hospital, Ethiopia. The study revealed that the proportion of hypertensive disorders of pregnancy was 3.56%, which was lower than the study conducted in Tikur Anbessa Hospital, Jimma University Specialized Hospital and Debre Berhan Referral Hospital [3, 5, 6, 10]. The reason might be due to the development of awareness creation made on controlling danger signs of maternal health by extension health workers in the current study than earlier study in a rural area.

This study showed that the extreme ages of reproductive years were found to be risk factors for hypertension during pregnancy with high incidence rates in old ages of greater than 35 years in comparison with the age range of 25–29 years. Concerning the current study, a hospital-based cross-sectional study conducted in Dassie Referral Hospital [11] and in Derashe, woreda [7] in Ethiopia reported that late age 30 years in some cases and age greater than 35 years in most cases were significantly associated with Hypertensive disorders of pregnancy.

About two folds of cases of HDP (64.8%) were living in a rural area comparing to urban residence for this study. Then the rural residential area was found to be one of the risk factors of HDP.

This finding was similar to a study done in Jimma hospital of a similar country [5].

In the current study, those women with prim gravida pregnancies had 3.40 times higher odds of developing hypertension disorders as compared with their counterparts. Also, the occurrence of HDP was reported more serious in prim gravida mothers of case groups than the control group [12]. This is maybe because getting pregnancy for the first time likely induces psychological stress and physical boredom that make women at risk of the development of HDP.

In this study, women with previous abortions had 4.40 times higher odds of more likely to develop hypertensive disorders than with no previous abortion. Which is inconsistent with the current findings. For instance, a study conducted in Iran reported a noticeable effect of the history of abortion on increasing the risk of mild preeclampsia [13]. further noted that there was no significant difference in the incidence of preeclampsia between women with no history of previous abortion and term pregnancy and women who had previous preterm birth [14, 15].

This study indicated that twin pregnancies had more than three folds of developing hypertension during pregnancy as compared with having singleton pregnancies. This result is in line with the research conducted in Northeastern Ethiopia [11]. This study has shown that lack of antenatal care had more likely associated with hypertension disorder during pregnancy. A similar finding was found in Egypt [16] in which preeclampsia was higher in women who had not ANC follow up. This could be due to women who had ANC follow up might get preventive measures for preeclampsia from health care providers during their ANC follow up.

The present study revealed that the positive previous history of preeclampsia was significantly associated with the development of hypertension. Women who had pre-existing hypertension were more likely to develop hypertensive disorders compared to women who had a negative family history of hypertension.

This study coincides with the findings reported as women presenting preeclampsia/eclampsia constituted a high-risk group for developing long term chronic hypertension [17]. Besides, there is consensus in the literature regarding the role of the previous history of preeclampsia as a contributing factor for preeclampsia [8, 10, 18].

In this study, a family history of hypertension had also a significant relationship with hypertensive disorders of pregnancy. Women who had a positive family history of hypertension were more likely to develop hypertensive disorders compared to women who had a negative family history of hypertension. More similar studies including in the Tigray region, Ethiopia revealed that a positive family history of chronic hypertension was a risk factor for HDP [8, 10, 15, 18–22].

From these findings, it seems that both maternal and fetal genes play a role in this syndrome. Therefore, for pregnant women with a family history of HDP, it should be monitored carefully both perinatally and in the postpartum period.

Another finding showed that gestational diabetes mellitus was significantly associated with hypertension disorders during pregnancy. It is supported by numerous studies that diabetes mellitus was considerable risk factors for the development of preeclampsia [10, 23–25].

In this study, diabetes Mellitus was found to be an important risk factor for developing HDP. It was 5.03 times higher for the positive history of diabetes mellitus. Thus, actions in public health focused to prevent these diseases are important to also prevent preeclampsia.

### Differences in pregnancy outcomes between women with and without HDP

Cases and control of this study found to have significant differences in maternal and perinatal comes. Accordingly, induced labor or cesarean sections (CS) was significantly higher in cases 81(40.7%) than in controls 8(2.0%). Besides, data obtained on the mode of delivery show that Cesarean Section was higher in cases than in controls were as normal spontaneous vaginal delivery and instrumental deliveries were more common in controls than in cases.

The difference between the prevalence of abruption placenta complication between women with and without HDP was found to be significant.

The magnitude of the abruption placenta was more than three-fold in cases compared to in controls. Corresponding to this finding, it was reported that placental abruption was a common complication of mothers experiencing any type of hypertension during pregnancy [26, 27].

A considerable number of studies have reported that preterm birth was significantly higher in women with HDP than without. For instance, a study conducted in China indicated that 29.36% of women who had HDP gave birth before 37 weeks of gestation than 6.78% of women without HDP [26]. Besides, a study in Portugal showed a statistically significant association between preterm delivery and severity of HDP [28].

Of course, it is very important to conducted further studies with adequate samples in different parts of our country to determine the magnitude of difference that case and control groups have on giving preterm births.

A study conducted in Mettu Karl Referral Hospital reported that 120.37 perinatal mortality per 1000 deliveries, 10.2% stillbirth rate,30.5% low birth weight, low 18.5% APGAR score and 31.4% preterm delivery outcomes in women with HDP [29].

Besides Tesfaye A and Tilahun M. indicated 21.2% of infants of women with HDP were admitted in a neonatal intensive care unit [30].

Regarding pregnancy complications, among women with HDP in this study, 24.6, 9.5, 8.0, 3.5, and 3.5% of them had developed complications of eclampsia, abruption placenta, DIC, acute renal failure, and pulmonary edema, respectively.

To this end, many studies reported similar findings [1, 6, 31–33]. The findings show that both maternal and fetal morbidity and mortality were higher in HDP. That is, maternal and perinatal complications women with HDP are common elsewhere in our world with a more severe rate in developing countries. Thus, improving antenatal care for pregnant mothers in our country is indispensable.

### Strength and limitation of the study

#### Strength

This study was done on the hypertensive disorders during pregnancy, which is one of the major maternal and perinatal cause of death. The use of a case-control study design helped to compare the effect of hypertension disorder between women with and without HDP.

#### Limitation

We utilized secondary data; which might be encountered to lack some of variables.

There were missed variables such as socio-demographic characteristic such as maternal education level, maternal weight, and height, smoking status of mothers.

## Conclusions and recommendations

### Conclusion

Women with hypertension during pregnancy have a greater risk of having adverse pregnancy outcomes as compared to normotensive pregnant women. Old age, rural residential area, being single, nulliparity, positive history of abortion, twin pregnancy, lack of ANC follows up, positive pre-existing hypertension, positive family history of hypertension and positive diabetes mellitus were identified as risk factors for developing hypertensive disorders of pregnancy.

### Recommendation

Based on the findings the following recommendations were given.

Strengthening ANC service to strengthen counseling and managing the complication early.

Strengthening neonatal intensive care unit (including expansion) in health facilities could be an important input in reducing neonatal complications.

## Data Availability

The data sets used and analyzed during the current study are available from the corresponding author on reasonable request.

## Abbreviations

ANC: Antenatal care
AOR: Adjusted odd ratio
C//S: Cesarean section
CI: Confident Interval
DIC: Disseminated intravascular coagulopathy
HDP: Hypertension disorders during pregnancy
OR: Odd Ratio
SD: Standard Deviation
